# Role of Blood Neurofilaments in the Prognosis of Amyotrophic Lateral Sclerosis: A Meta-Analysis

**DOI:** 10.3389/fneur.2021.712245

**Published:** 2021-10-06

**Authors:** Yan-ni Zhou, You-hong Chen, Si-qi Dong, Wen-bo Yang, Ting Qian, Xiao-ni Liu, Qi Cheng, Jiu-cun Wang, Xiang-jun Chen

**Affiliations:** ^1^Ministry of Education Key Laboratory of Contemporary Anthropology, Department of Anthropology and Human Genetics, School of Life Sciences, Fudan University, Shanghai, China; ^2^Department of Neurology, Huashan Hospital, Institute of Neurology, Fudan University and National Center Neurological Disorders, Shanghai, China; ^3^Department of Neurology, Ruijin Hospital Affiliated With the School of Medicine, Shanghai Jiao Tong University, Shanghai, China; ^4^Department of Dermatology, Huashan Hospital and Human Phenome Institute, Fudan University, Shanghai, China; ^5^Institute of Rheumatology, Immunology and Allergy, Fudan University, Shanghai, China

**Keywords:** amyotrophic lateral sclerosis, neurofilament, disease progression, meta-analysis, survival

## Abstract

**Background:** Neurofilaments in cerebrospinal fluid (CSF) and in blood are considered promising biomarkers of amyotrophic lateral sclerosis (ALS) because their levels can be significantly increased in patients with ALS. However, the roles of neurofilaments, especially blood neurofilaments, in the prognosis of ALS are inconsistent. We performed a meta-analysis to explore the prognostic roles of blood neurofilaments in ALS patients.

**Methods:** We searched all relevant studies on the relationship between blood neurofilament levels and the prognosis of ALS patients in PubMed, Embase, Scopus, and Web of Science before February 2, 2021. The quality of the included articles was assessed using the Quality in Prognosis Studies (QUIPS) scale, and R (version 4.02) was used for statistical analysis.

**Results:** Fourteen articles were selected, covering 1,619 ALS patients. The results showed that higher blood neurofilament light chain (NfL) levels in ALS patients were associated with a higher risk of death [medium vs. low NfL level: HR = 2.43, 95% CI (1.34–4.39), *p* < 0.01; high vs. low NfL level: HR = 4.51, 95% CI (2.45–8.32), *p* < 0.01]. There was a positive correlation between blood phosphorylated neurofilament heavy chain (pNfH) levels and risk of death in ALS patients [HR = 1.87, 95% CI (1.35–2.59), *p* < 0.01]. The levels of NfL and pNfH in blood positively correlated with disease progression rate (DPR) of ALS patients [NfL: summary *r* = 0.53, 95% CI (0.45–0.60), *p* < 0.01; pNfH: summary *r* = 0.51, 95% CI (0.24–0.71), *p* < 0.01].

**Conclusion:** The blood neurofilament levels can predict the prognosis of ALS patients; specifically, higher levels of blood neurofilaments are associated with a greater risk of death.

## Introduction

Amyotrophic lateral sclerosis (ALS) is a neurodegenerative disorder that selectively causes the degeneration of upper and lower motor neurons, resulting in progressive muscle wasting and weakness (1). Patients often die of respiratory failure 3–5 years after disease onset (2, 3). Riluzole and edaravone are the only drugs approved by the US Food and Drug Administration (FDA) for the treatment of ALS; both drugs have limited efficacy and are only effective in some patients (4–6).

Up to now, no fluid biomarkers in ALS have entered clinical practice, but they are urgently needed. Neurofilaments are potential biomarkers in ALS; they are considered promising and useful biomarkers for the diagnosis of ALS (7). However, their prognostic roles in ALS are still not clear. Neurofilaments are the intermediate fibers of nerve cells; they can be divided into three subunits: neurofilament light chain (NfL), neurofilament medium chain, and neurofilament heavy chain (NfH) (8). In a mouse model of ALS, researchers found that neurofilament subunits were associated with the pathogenesis of ALS (9, 10). It has been shown that patients with ALS have elevated neurofilament levels compared to control groups, both in cerebrospinal fluid (CSF) and in blood (11–14). However, the roles of neurofilaments, especially blood neurofilaments, in the prognosis of ALS are inconsistent (15–19). NfH is the most widely phosphorylated protein in the human brain; therefore, phosphorylated NfH (pNfH) is more stable and less susceptible to degradation by proteases, representing a steady and reproducible biomarker in consecutive measurements (20, 21). It has been proposed that pNfH correlates with the extent of motor neuron degeneration (22). Therefore, pNfH is commonly used to replace NfH in the study of potential ALS biomarkers.

The neurofilaments in CSF are considered potential predictive factors of ALS patients' prognosis (23–25). However, CSF collection requires lumbar puncture, and samples are relatively difficult to collect. By contrast, with the development of detection technology, the accuracy of neurofilament detection in blood has improved, and venous blood drawing is relatively simple, economical, non-invasive, repeatable, and acceptable. In addition, serum neurofilaments have been reported to strongly correlate with CSF neurofilaments (16, 18). Therefore, blood is more likely to be used as a test sample in clinical and pharmaceutical studies.

Thus, we performed a meta-analysis of published studies to explore the relationship between blood neurofilaments levels and indicators related to ALS progression and prognosis.

## Methods

### Search Strategy

The meta-analysis followed the guidelines of PRISMA (Preferred Reporting Items for Systematic Reviews and Meta-Analyses). Four computerized databases, including PubMed, Embase, Scopus, and Web of Science, were used to collect all studies on the association between blood neurofilament levels and ALS. We searched the databases for relevant English-language literature before February 2, 2021. The search string was built as follows: (((((“Amyotrophic Lateral Sclerosis”) OR (“ALS”)) OR (“Charcot Disease”)) OR (“Lou Gehrig Disease”)) OR (“Lou Gehrig's Disease”)) OR (“Motor Neuron Disease”) AND (((“Blood”) OR (“Plasma”)) OR (“Serum”))) AND ((((“Neurofilaments”) OR (“Neurofilament”)) OR (“NfL”)) OR (“Phosphorylated NfH”)). To avoid missing relevant literature, the electronic database search was supplemented by a manual search of the reference lists of the included articles. Two researchers (Yanni Zhou and Youhong Chen) searched the literature independently. Any disagreement was resolved by discussion until consensus was reached, or a third researcher would make the decision. This meta-analysis was registered with PROSPERO (registration number: CRD42020203464).

### Selection and Exclusion Criteria

**Figure 1:**
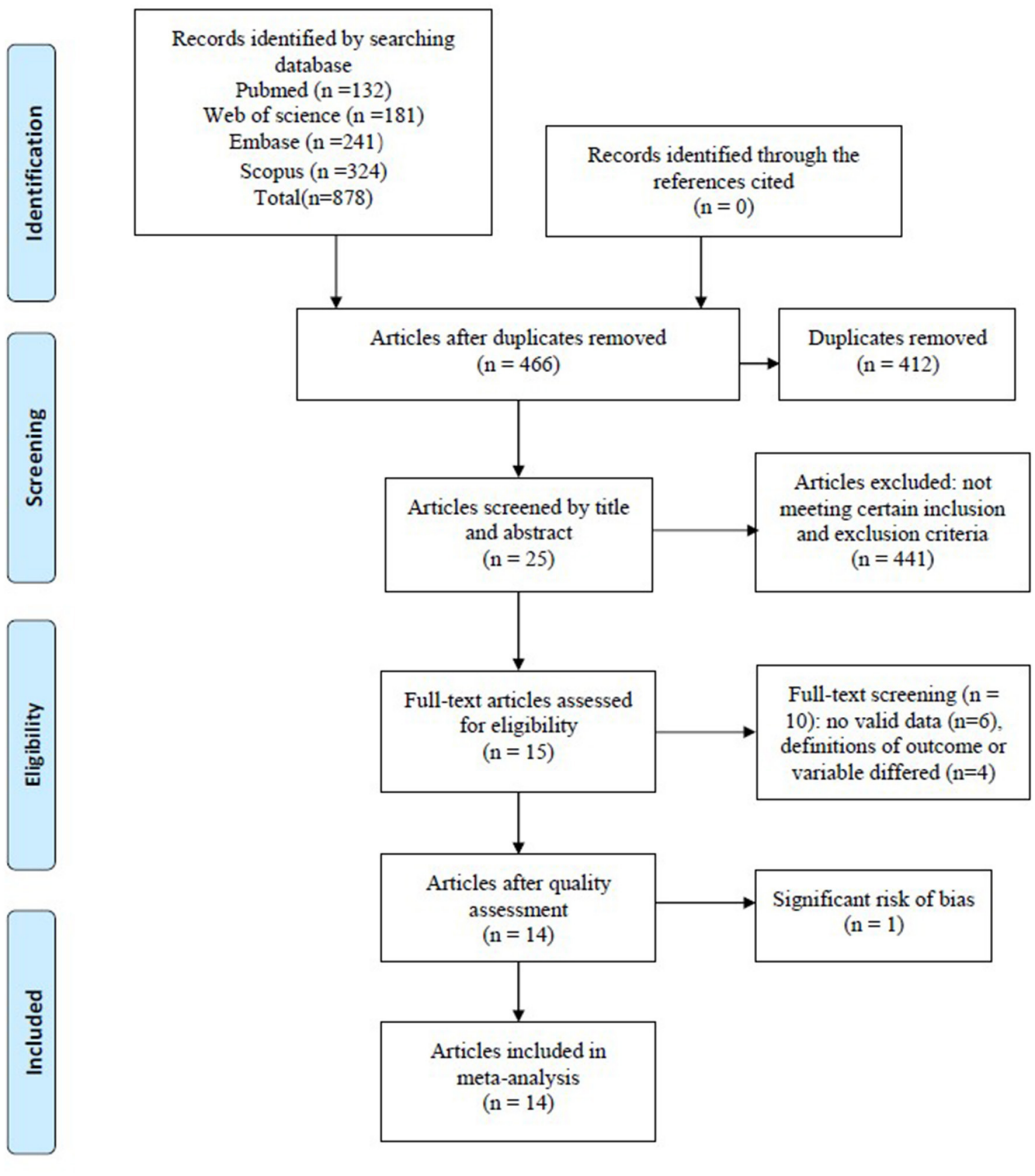
Flowchart of document retrieval.

Figure 1 shows the flowchart of the selection process.

Inclusion criteria were as follows: (1) publication language is English; (2) original text is available; (3) ALS patients were aged 18 years and over; (4) Awaji criteria or the El Escorial or revised El Escorial criteria are used to diagnose ALS; and (5) the role of blood neurofilaments as biomarkers in ALS is studied.

Exclusion criteria were as follows: (1) there were other severe diseases that could affect the ALS phenotype (e.g., frontotemporal dementia or cerebral injury); (2) lack of a control group; (3) article is a case report, meta-analysis, review, letter, animal study, or *in vitro* study; (4) relevant data are unavailable; and (5) definitions of outcome or variables differed significantly from most studies.

### Quality Assessment

The Quality in Prognosis Studies (QUIPS) scale was used to evaluate the quality of the included studies. This scale included questions related to areas that can inform judgments about the risk of bias in prognostic research (26). The assessment was divided into six items: study participation, study attrition, prognostic factor measurement, outcome measurement, study confounding, statistical analysis, and reporting. Each item was rated as low risk, moderate risk, or high risk. The grade evaluation of each article was carried out independently by two researchers (Siqi Dong and Wenbo Yang), and any inconsistency was resolved by discussion or by a third researcher. Since the QUIPS scale does not provide a uniform, full-text risk-assessment standard, only the risk grades for each item in the included studies were given in this meta-analysis. Studies with a significantly higher risk of bias were excluded.

### Data Extraction

Two authors (Yanni Zhou and Youhong Chen) extracted data independently; any disagreement was resolved by reexamination and discussion or by a third author. The following data were extracted: author name, year of publication, country where the study was conducted, follow-up duration of ALS patients, number of ALS patients and controls included in the study, hazard ratio (HR), 95% confidence intervals (95% CI), levels of blood neurofilaments in all subjects, measurement method, and control factors in Cox multivariate regression analysis.

### Statistical Analysis

We planned to compare blood neurofilament levels between ALS patients and control subjects, and we extracted standardized mean differences with a 95% CI. HRs with 95% CIs were collected to evaluate the association between blood neurofilament levels and mortality risk in ALS patients. Correlation coefficients (*r*) were recorded to assess the relationship between blood neurofilament levels and the rate of disease progression in ALS patients. Heterogeneity analysis was assessed using the Cochran's *Q* test and the *I*^2^ statistic. *I*^2^ > 50% or *p* < 0.1 represented substantial heterogeneity; then, a random-effects model was chosen. Otherwise, a fixed-effects model was chosen. Publication bias was assessed qualitatively by the funnel plot method and quantitatively by Egger's test. To assess the effect of each study on the pooled estimate, sensitivity analysis was done by removing each study by turns and switching the effects model. R (version 4.02) was used for statistical analysis. In addition, Spearman's correlation coefficient extracted from the data had to be transformed as follows (formulas 1–5). Spearman's correlation coefficient was converted into Pearson's correlation coefficient using formula (1) (27):

(1) *r*_p_ = 2sin($r_{\text{s}}\;*\;\frac{\pi }{6}$);
(2) Fisher's *Z* = 0.5 * ln$\frac{1+r}{1-r}$;
(3) *V*_z_ = $\frac{1}{n-\;3}$;
(4) *S*_E_ = $\sqrt{v_{z}}$;
(5) Summary *r* = $\frac{e^{2z}-1}{e^{2z}+1}$ (*Z* is summary Fisher's *Z*).

## Results

### Search Results and Study Characteristics

**Figure 2:**
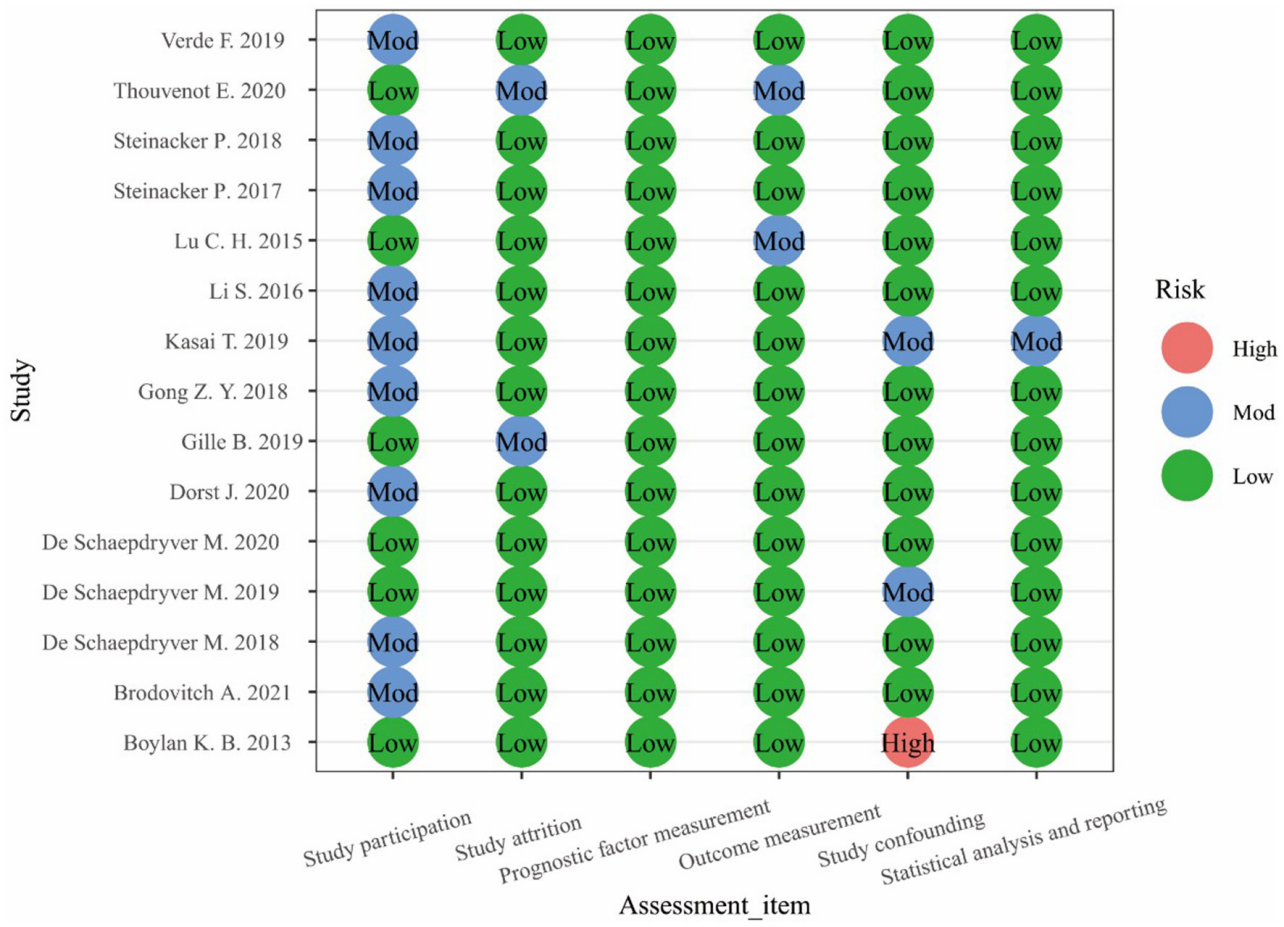
Quality evaluation chart.

We searched relevant articles in PubMed (*n* = 132), Web of Science (*n* = 181), Embase (*n* = 241), and Scopus (*n* = 324); a total of 878 relevant articles were retrieved. After excluding 412 duplicate articles, the remaining 466 articles were screened by title and abstract, and 441 articles that did not meet the requirements were excluded. Then, the remaining 25 articles were screened by full-text reading; 10 articles among them did not meet the requirements and were excluded. The remaining 15 articles were assessed for quality, and one of the articles (28) was excluded because it had a significant risk of bias in three items: study participation, study confounding, statistical analysis, and reporting. Finally, 14 articles were included for data extraction, representing a total of 1,619 ALS patients. The results are shown in Figure 2. In the actual review process, we found that different researchers used different blood components (plasma/serum) to measure neurofilament subunit levels (NfL/pNfH), but there were relatively few studies on neurofilament levels in plasma. Therefore, plasma and serum were not distinguished in this meta-analysis. Quality assessment results showed that one study team used different sample sizes from the same cohort to do relevant analyses (14, 29); therefore, we chose the article with the largest sample size and the research purpose more consistent with the theme of this paper for data extraction (14). One study used converted data for analysis (30). In order to exclude the influence of data conversion on the results, we did not include it in the final data extraction. In addition, the definition of disease progression rate (DPR) in the included studies was relatively consistent—that is, DPR = (48-ALSFRS-R at “time of diagnosis”)/duration from onset to diagnosis (months) (31). However, the definition of DPR in one study was significantly different from the other studies (15), and the data from that study were not used in the analysis of the relationship between blood neurofilaments and ALS DPR. After quality evaluation, one study with a high risk of bias was excluded (28).

### Blood Neurofilaments and Survival

#### NfL and Survival

In the process of data extraction, we found that only four articles exploring the relationship between serum NfL and survival were qualified. One article reported studies of two different cohorts (16). There were two articles grouping ALS patients by the tertiles of NfL level (16, 18). To better describe the groupings, according to the researchers' grouping characteristics, patients with the lowest, intermediate, and highest tertile NfL levels were referred to as the low NfL group, middle NfL group, and high NfL group, respectively. There was only one qualified article exploring the relationship between plasma NfL and survival, and it was a subcolumn study in the article (16). Therefore, we did not distinguish blood sample types. Finally, two articles with three studies were included in the analysis (16, 18).

**Figure 3:**
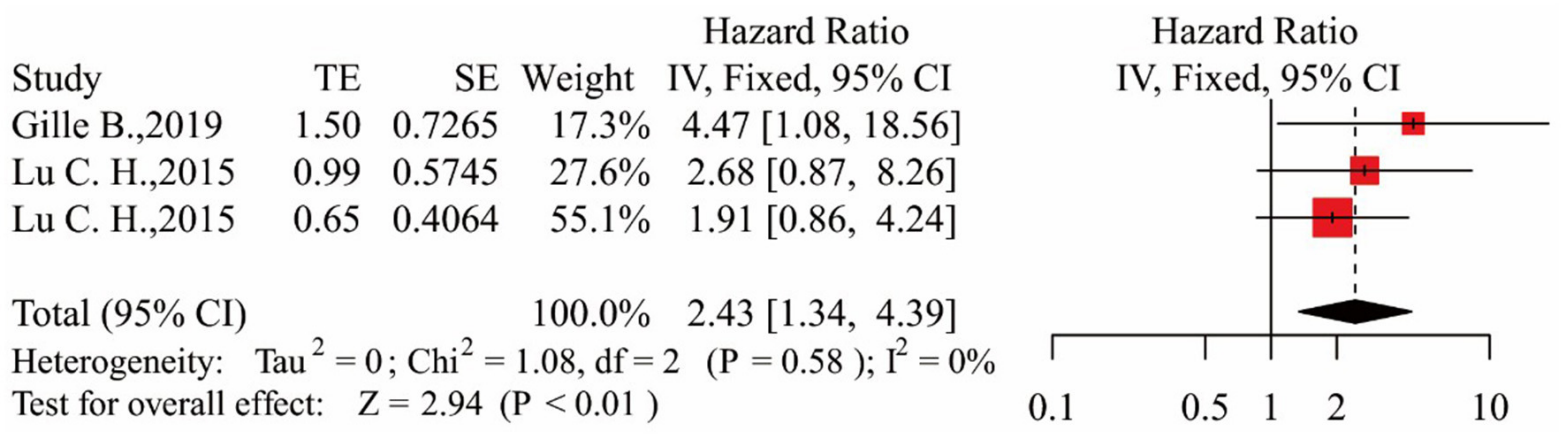
Forest map showing the relationship between NfL levels and survival (middle NfL group relative to low NfL group).

**Figure 4:**
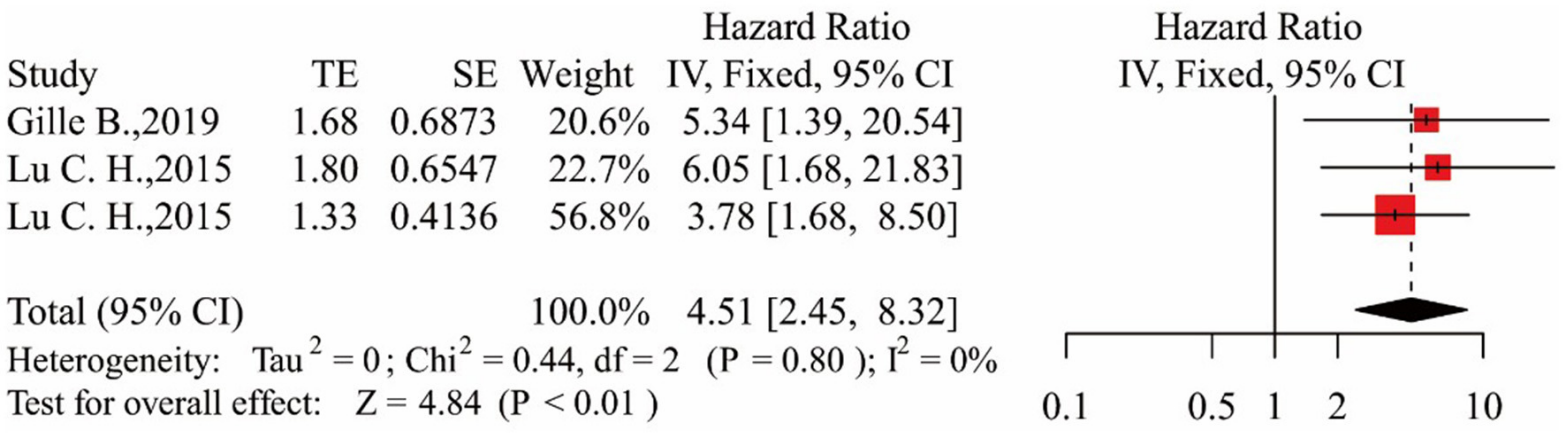
Forest map showing the relationship between NfL levels and survival (high NfL group relative to low NfL group).

In the three studies, the results showed that patients in the middle NfL group had a higher risk of death than those in the low NfL group [HR = 2.43, 95% CI (1.34–4.39), *p* < 0.01]. The results are shown in Figure 3. Patients in the high NfL group also had a higher risk of death than patients in the low NfL group [HR = 4.51, 95% CI (2.45–8.32), *p* < 0.01]. The results are shown in Figure 4. The Egger's test results were all *p* > 0.05; no obvious bias was found in the funnel plot, and the sensitivity analysis results showed that the relationship between NfL levels and survival was stable (Supplementary Figures 1–4). The remaining two included articles exploring the relationship between serum NfL and ALS patients' survival did not group ALS patients by the tertiles of NfL levels. De Schaepdryver et al. (32) found that HR of serum NfL was 2.21 [95% CI (1.51–3.24), *p* < 0.0001], and Thouvenot et al. (19) found that patients with serum NfL levels ≥ 71.2 pg/ml had a higher risk of death [HR = 4.7, 95% CI (3.0–7.4), *p* < 0.0001]. In conclusion, higher levels of NfL in ALS patients' blood were associated with a higher risk of death.

#### pNfH and Survival

**Figure 5:**
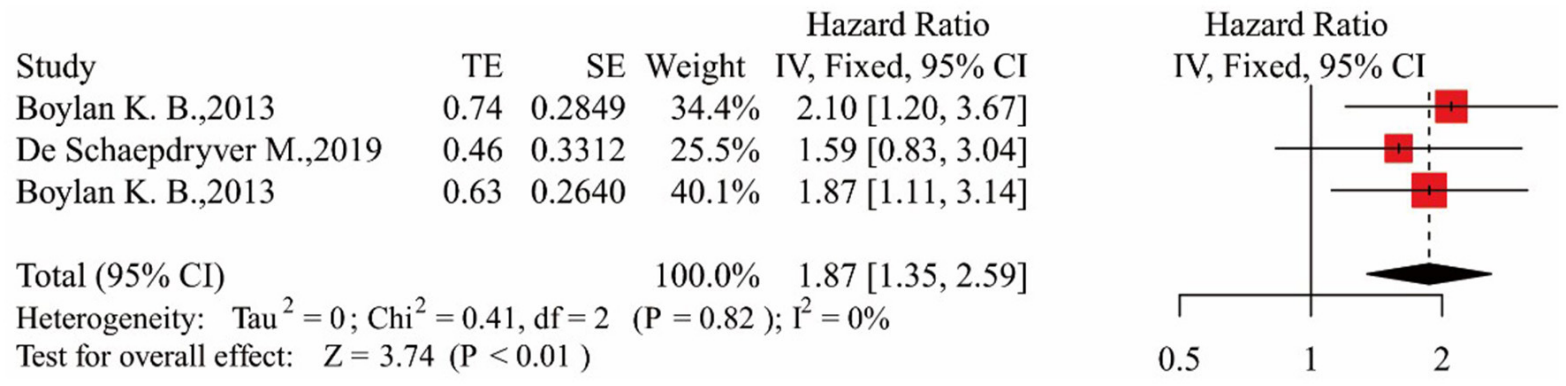
Forest map showing the relationship between pNfH levels and survival.

Two articles exploring the relationship between blood NfH and survival were included as eligible articles (15, 17). In one of them (15), researchers took measurements in both plasma and serum. The results showed that ALS patients had higher blood NfH levels and a higher risk of death compared with the control group [HR = 1.87, 95% CI (1.35–2.59), *p* < 0.01]. The results are shown in Figure 5. The Egger's test result was *p* > 0.05; no obvious bias was found in the funnel plot, and the sensitivity analysis results showed a stable relationship between pNfH levels and survival (Supplementary Figures 5, 6).

### Blood Neurofilaments and DPR

#### NfL and DPR

**Figure 6:**
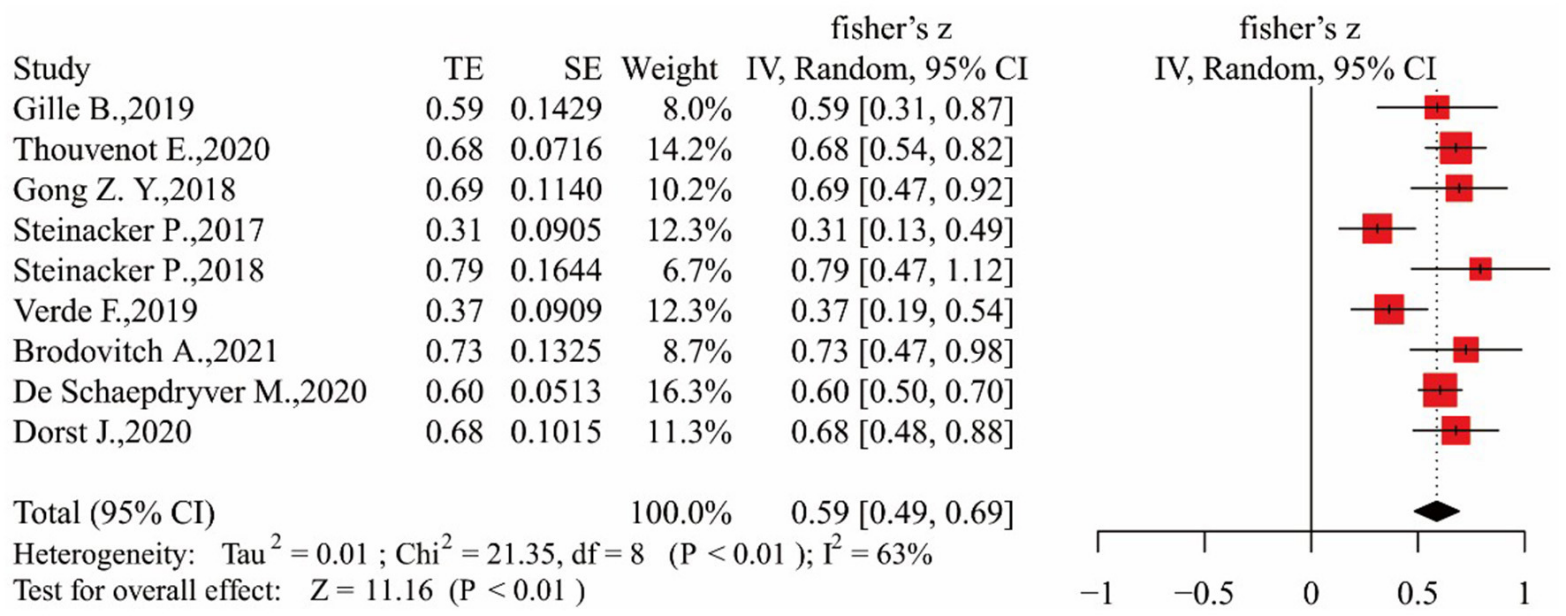
Forest map showing the relationship between NfL levels and DPR.

We found that authors had used a consistent definition of DPR; that is, DPR = (48-ALSFRS-R at “time of diagnosis”)/duration from onset to diagnosis (months) (31). During the data extraction process, 10 qualified articles were included to study the relationship between serum NfL and DPR (14, 16, 18, 19, 29, 32–36). We excluded one of these studies (16) because it only studied the relationship between neurofilaments and DPR in patients with rapid progression. There were no qualified studies on the measurement of neurofilaments in plasma. Finally, nine articles were included for data analysis (14, 18, 19, 29, 32–36). The results are shown in Figure 6 (*I*^2^ = 63%), and a random-effect model was employed to calculate the outcomes. As shown in Figure 6, summary Fisher's *Z* value was 0.59, and after conversion according to formula (5), summary *r* was 0.53 [95% CI (0.45–0.60)]. The Egger's test result was *p* > 0.05; no obvious bias was found in the funnel plot, and the sensitivity analysis results showed a stable relationship between NfL levels and DPR (Supplementary Figures 7, 8).

#### pNfH and DPR

**Figure 7:**
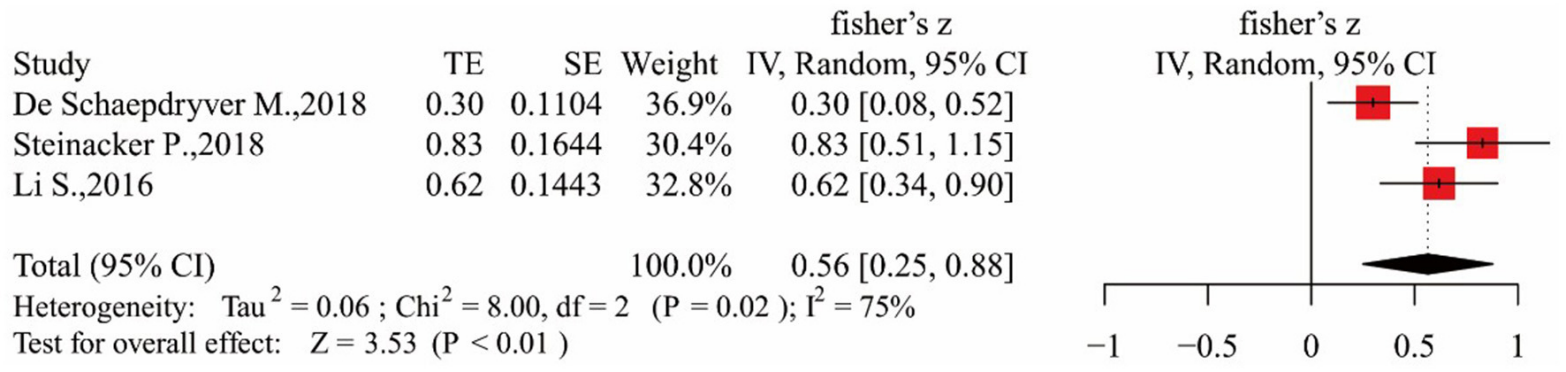
Forest map showing the relationship between pNfH levels and DPR.

We included three eligible studies exploring the relationship between blood pNfH and DPR (29, 37, 38). However, there was great heterogeneity among these studies (*I*^2^ = 75%, *p* < 0.05), so we used a random-effects models for calculation. The results are shown in Figure 7. Summary *r* was 0.51 after conversion [95% CI (0.24–0.71), *p* < 0.01], and Egger's test result was *p* < 0.05, indicating that there was bias. Yet, the sensitivity analysis showed a stable relationship between pNfH levels and DPR (Supplementary Figures 9, 10).

## Discussion

Many metabolites have been considered to have potential predictive power in ALS patients' prognosis. For example, serum creatinine levels were reported to positively correlate with ALS patients' survival (39), and decreased blood albumin levels could increase the risk of ALS patients' death (40). Neurofilaments are highly expressed in neurons; they are released into CSF when axons disintegrate, and then neurofilaments enter blood circulation (8). Neurofilament subunits play important roles in neuronal inclusion body formation, axonal mutagenesis, and neuronal death, which are all pathological characteristics of ALS (41). As important pathological markers of ALS, neurofilaments have great potential as prognostic biomarkers of ALS. Most studies have shown higher levels of both NfL (12, 16, 19) and pNfH (38, 42, 43) in ALS patients compared with controls. However, conclusions about the role of blood neurofilaments in the prognosis of ALS are inconsistent. Therefore, it is necessary to further investigate the role of neurofilament levels in the prognosis of ALS.

In fact, we found that there were few relevant and qualified studies that could be included in our analysis; even fewer articles could be included after serum and plasma were distinguished. Therefore, we did not distinguish between serum and plasma. The results of our meta-analysis indicated that patients with high blood NfL levels had a higher risk of death, and the risk of death increased with increasing NfL levels. These findings are also consistent with studies that did not group ALS patients by the tertiles of NfL levels (19, 32). The results of publication bias analysis and sensitivity tests also supported the stability of this conclusion.

The results of studies on the relationship between ALS patients' survival and blood NfL levels showed a positive correlation between blood NfL and DPR, indicating that higher NfL levels were associated with a higher risk of death. The results of publication bias analysis and sensitivity test indicated that the results were reliable. The correlation coefficient between blood NfL levels and DPR was 0.53, which means the correlation between blood NfL levels and DPR at baseline could be up to 53%. This conclusion is consistent with the above conclusion that higher NfL levels are associated with a higher risk of death in ALS patients; hence, the higher the blood NfL levels in ALS patients, the worse their prognosis.

Blood pNfH also positively correlated with DPR (summary *r* = 0.51), which means that higher pNfH levels in ALS patients were associated with a faster speed of disease progression. Although sensitivity analysis showed that the results were stable, the funnel plot and Egger's test results showed that the included articles were at risk of publication bias. It was speculated that the first reason was that there were few articles included. Second, the correlation coefficient value of each research result was quite different because correlation coefficient is related to the research design, sample size, and statistical analysis method of the actual research. Third, the results of the included studies were all significant, which could lead to bias. Despite publication bias, in general, blood pNfH positively correlated with DPR, and this relationship may reflect the real relationship of the two. This result is consistent with the conclusion that higher pNfH levels are associated with worse prognosis for ALS patients.

With the development of ultrasensitive stable assays, blood neurofilaments can be measured with a single-molecule array (Simoa) with high sensitivity. However, most of the included studies used enzyme-linked immunosorbent assay (ELISA) or electrochemiluminescence immunoassay (ECLI) to detect neurofilaments (shown in Supplementary Tables 1–4). Simoa allows the detection of clinically relevant proteins in serum at concentrations (<10^−15^ M) much lower than those measured by conventional ELISA (44), and it is also more sensitive than ECLI (45). The use of Simoa has become increasingly widespread in recent years, but there were few qualified articles using Simoa in this study (shown in Supplementary Tables 1–4), so we cannot even determine whether Simoa outperforms the other two methods. Nevertheless, we believe that since ELISA and ECLI can show the positive relationship between blood neurofilaments and ALS prognosis and survival, Simoa would be more likely to give a positive result due to its high detection sensitivity.

In summary, although there are relatively few studies on the relationship between blood neurofilament levels and the prognosis of ALS, blood neurofilament levels (NfL/pNfH) may be good predictive biomarkers of ALS patients; that is, higher blood neurofilament levels may be linked to faster DPR and higher risk of death in ALS patients. Although neurofilaments are not specific markers of ALS (e.g., neurofilaments are also promising biomarkers for multiple sclerosis, Alzheimer's disease, and Charcot–Marie–Tooth disease) (46–48), the predictive effect of neurofilaments on the prognosis of ALS patients suggests to some extent that the pathophysiological mechanism of ALS may be related to changes in the function and concentration of neurofilaments. Besides, neurodegenerative diseases are complex, and biomarkers are just one of the tools to help with diagnosis and treatment. We need to combine many aspects, including clinical features, laboratory results, and other useful information, to facilitate disease diagnosis and treatment.

## Conclusion

Our results showed that blood neurofilament levels can predict the prognosis of ALS patients, meaning higher levels of blood neurofilaments are associated with a higher DPR and a greater risk of death in ALS patients. These results contribute to the evidence that blood neurofilaments may be reliable biomarkers of ALS. We believe that more studies are needed to confirm these results.

## Data Availability Statement

The original contributions presented in the study are included in the article/Supplementary Material, further inquiries can be directed to the corresponding author/s.

## Author Contributions

All authors listed have made a substantial, direct and intellectual contribution to the work, and approved it for publication.

## Funding

This study was supported by the National and Provincial Multidisciplinary Cooperation in the Diagnosis and Treatment of Major Disease Capacity Improvement Project (Shanghai Municipal Health Commission) and the Shanghai Municipal Science and Technology Major Project (2017SHZDZX01) and ZJLab.

## Conflict of Interest

The authors declare that the research was conducted in the absence of any commercial or financial relationships that could be construed as a potential conflict of interest.

## Publisher's Note

All claims expressed in this article are solely those of the authors and do not necessarily represent those of their affiliated organizations, or those of the publisher, the editors and the reviewers. Any product that may be evaluated in this article, or claim that may be made by its manufacturer, is not guaranteed or endorsed by the publisher.
