# Supplementary material for: Role of Blood Neurofilaments in the Prognosis of Amyotrophic Lateral Sclerosis: A Meta-Analysis
Source: Front Neurol. 2021 Oct 6;12:712245. doi: 10.3389/fneur.2021.712245 (PMC8526968; doi:10.3389/fneur.2021.712245)
Supplement: Supplementary file 2 [file Data_Sheet_2.docx]

Supplementary Material


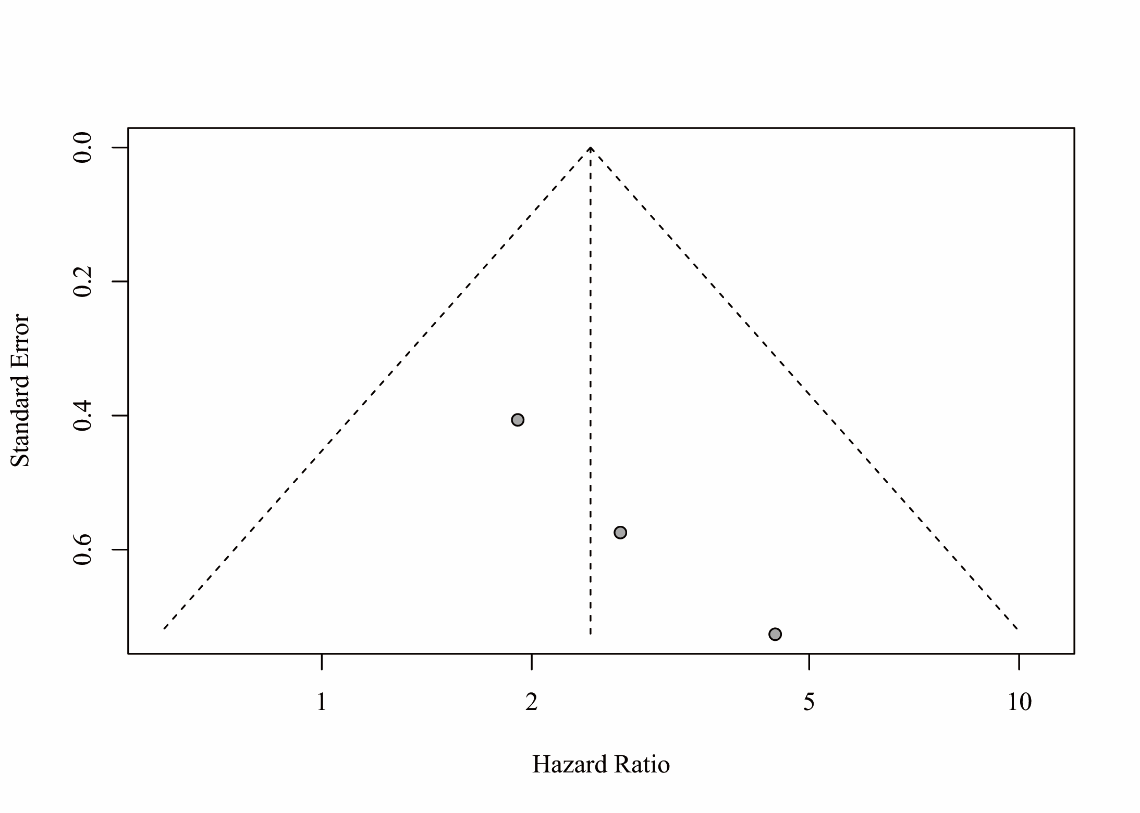


Figure 1. Publication bias plot about NfL levels and survival

(middle NfL group relative to low NfL group)


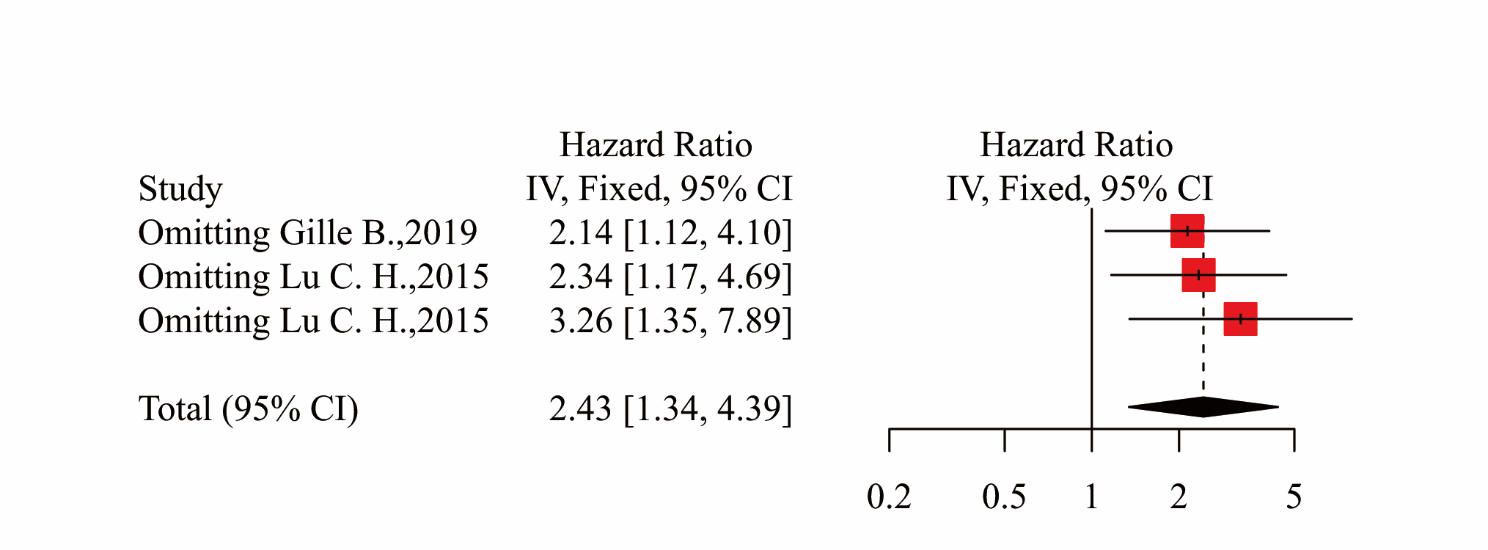


Figure 2. Sensitivity analysis diagram showing the relationship between NfL levels and survival

(middle NfL group relative to low NfL group)


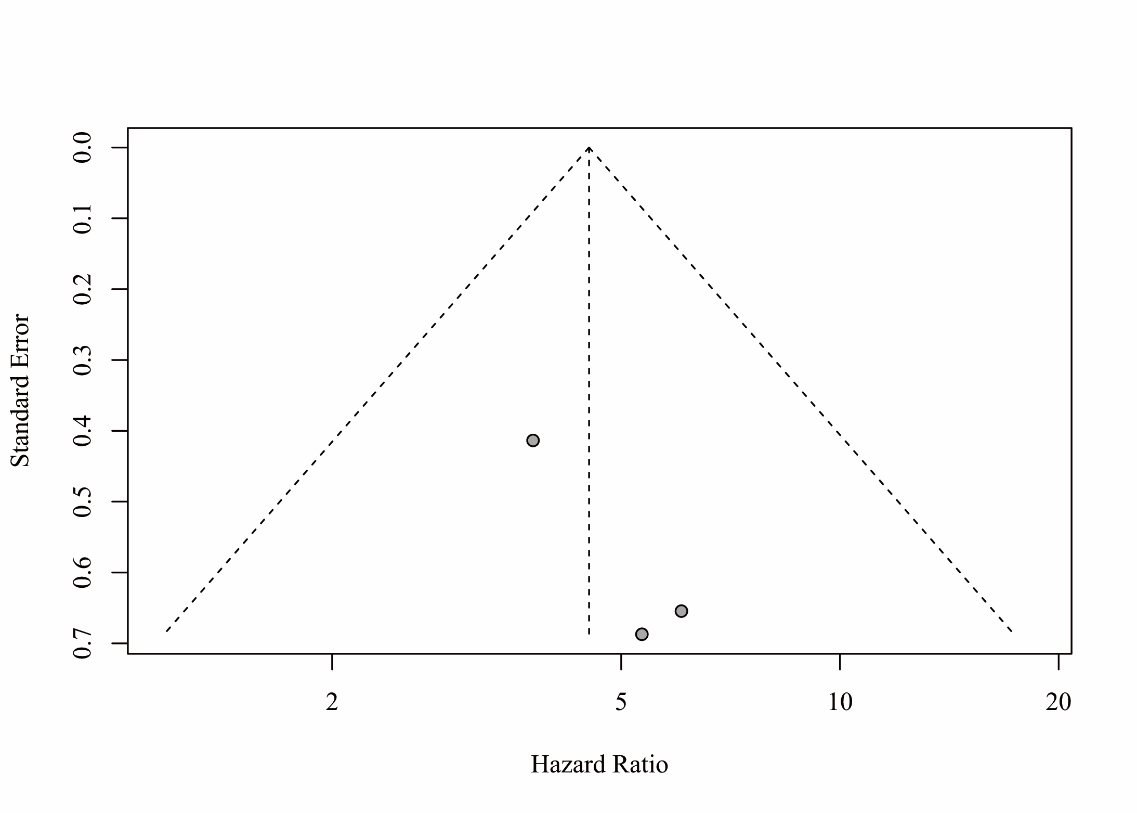


Figure 3. Publication bias plot about NfL levels and survival

(high NfL group relative to low NfL group)


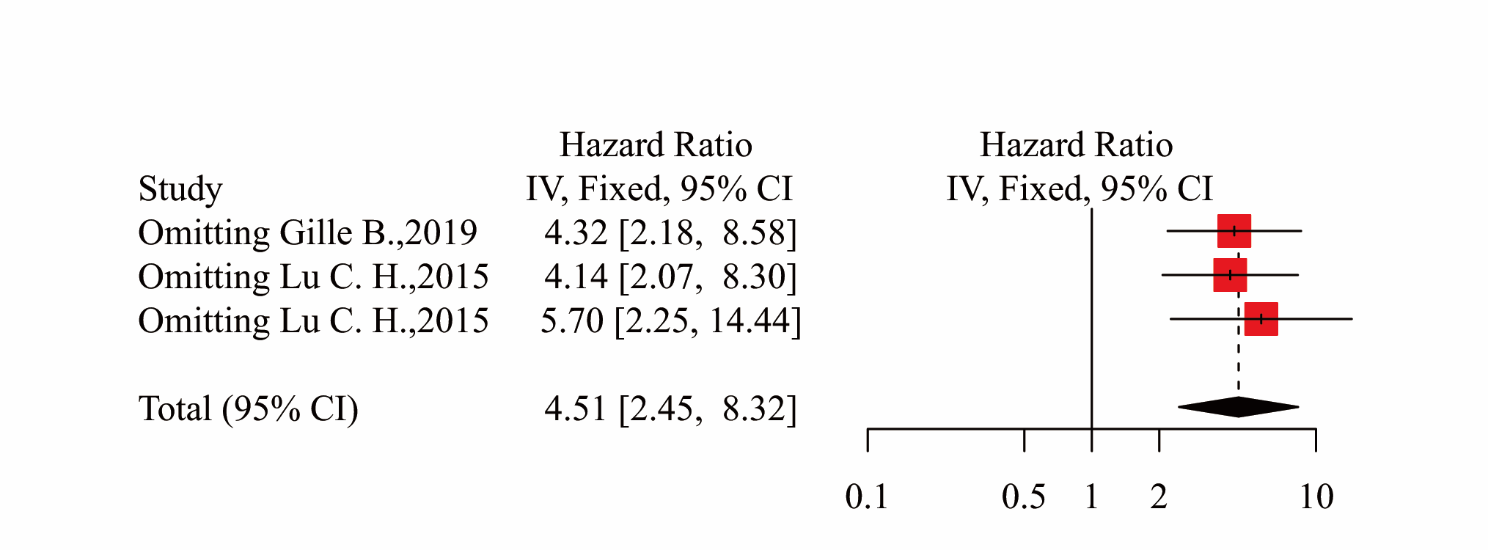


Figure 4. Sensitivity analysis diagram showing the relationship between NfL levels and survival

(high NfL group relative to low NfL group)


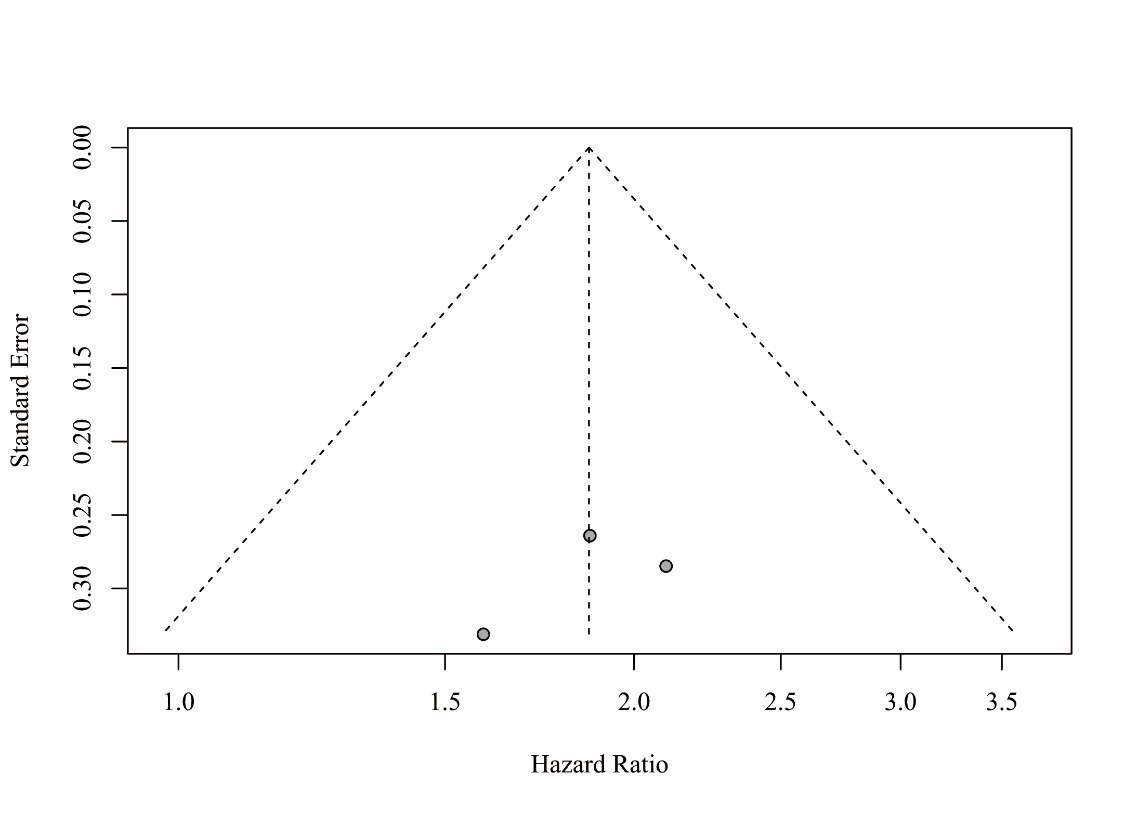


Figure 5. Publication bias plot about pNfH levels and survival


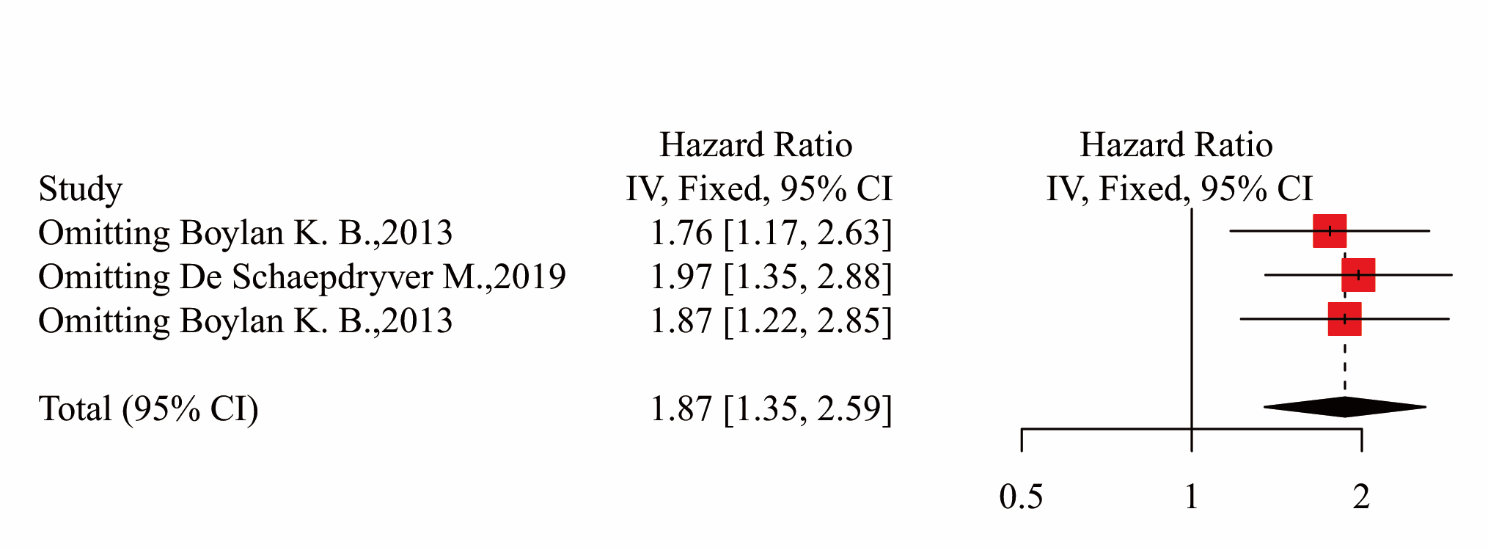


Figure 6. Sensitivity analysis diagram showing the relationship between pNfH levels and survival


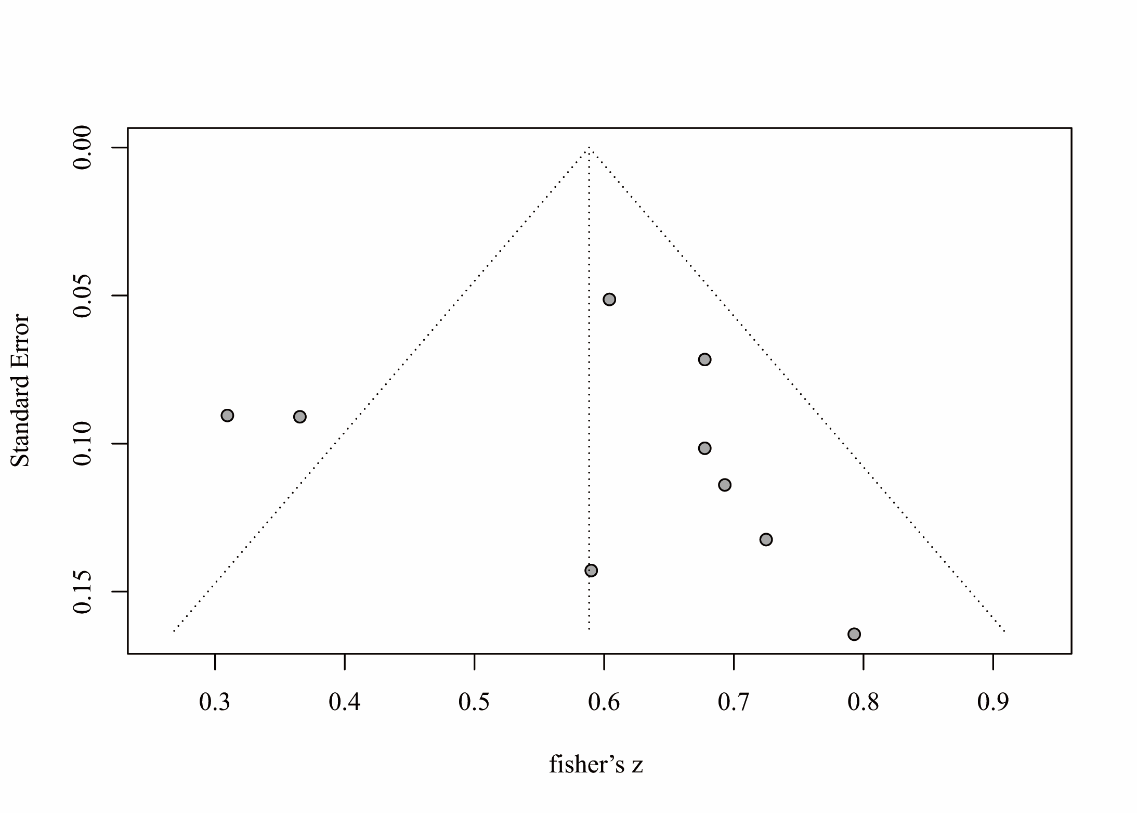


Figure 7. Publication bias plot about NfL levels and disease progression rate


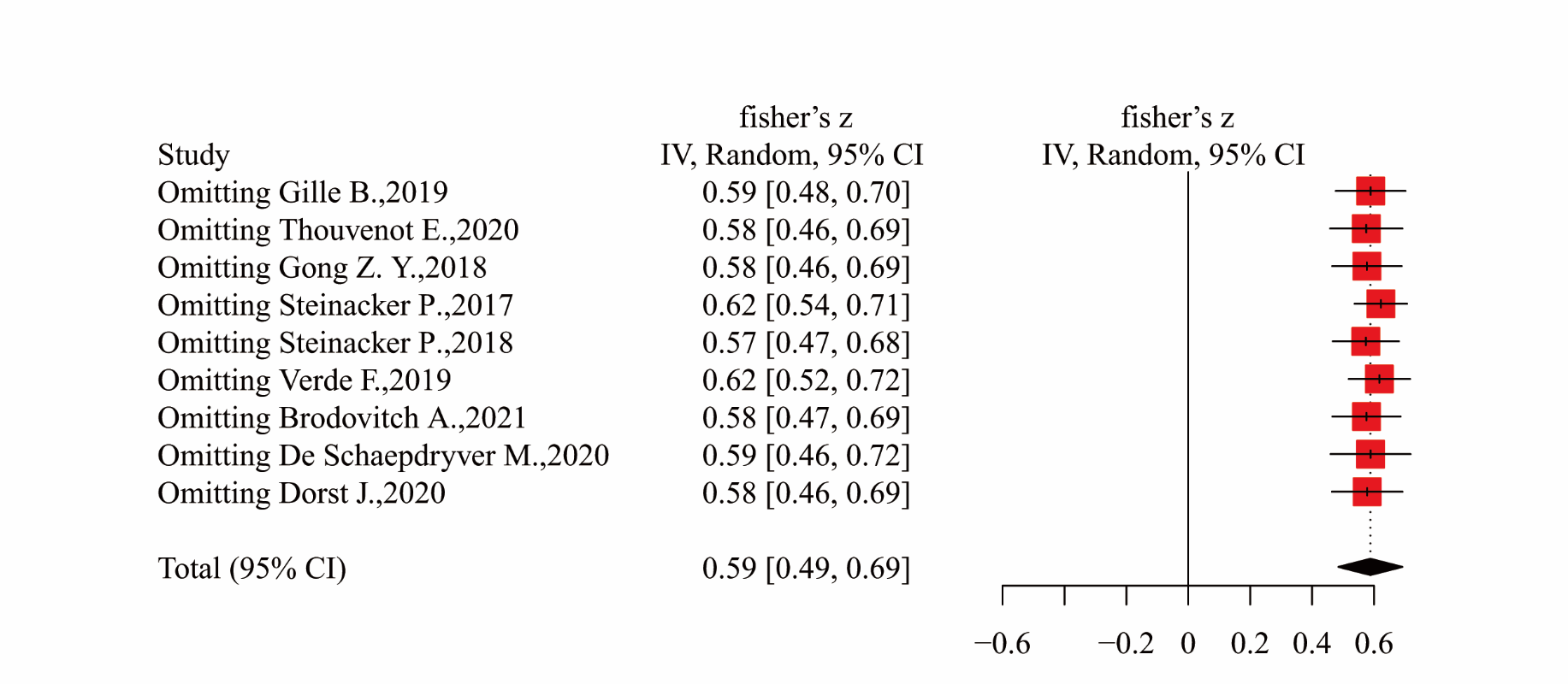


Figure 8. Sensitivity analysis diagram showing the relationship between NfL levels and disease progression rate


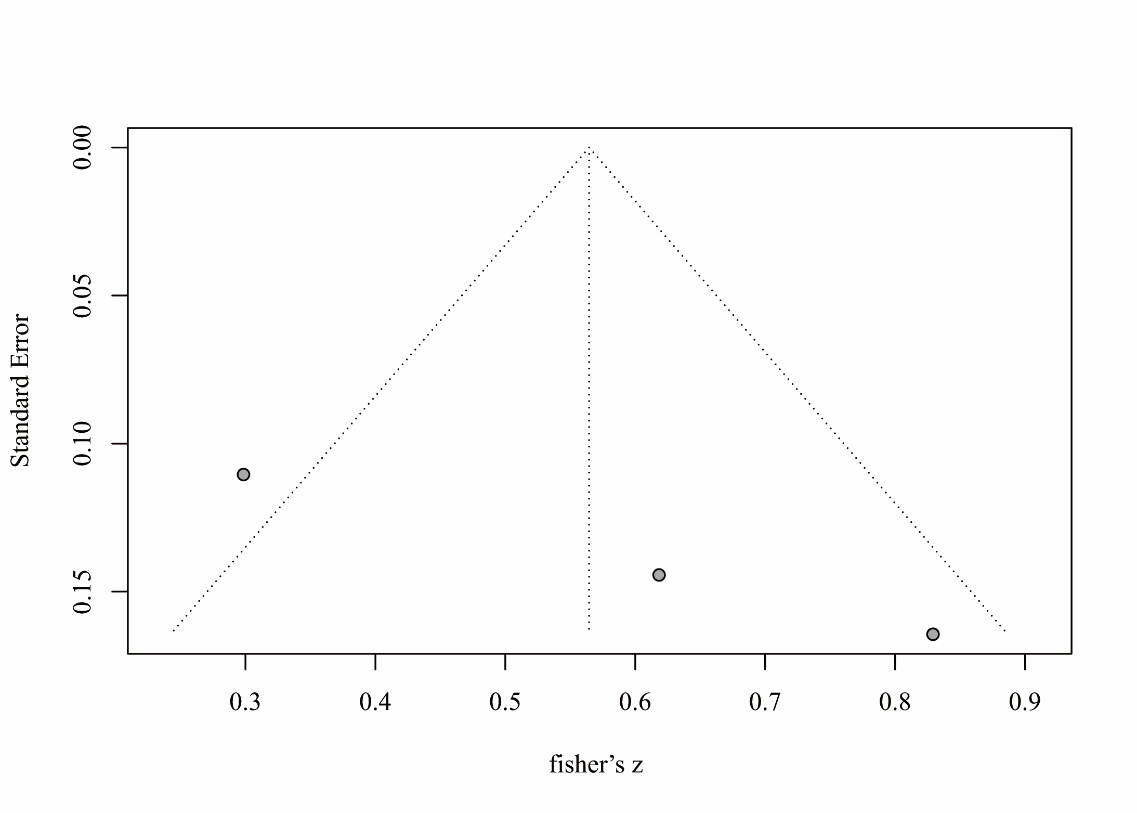


Figure 9. Publication bias plot about pNfH levels and disease progression rate


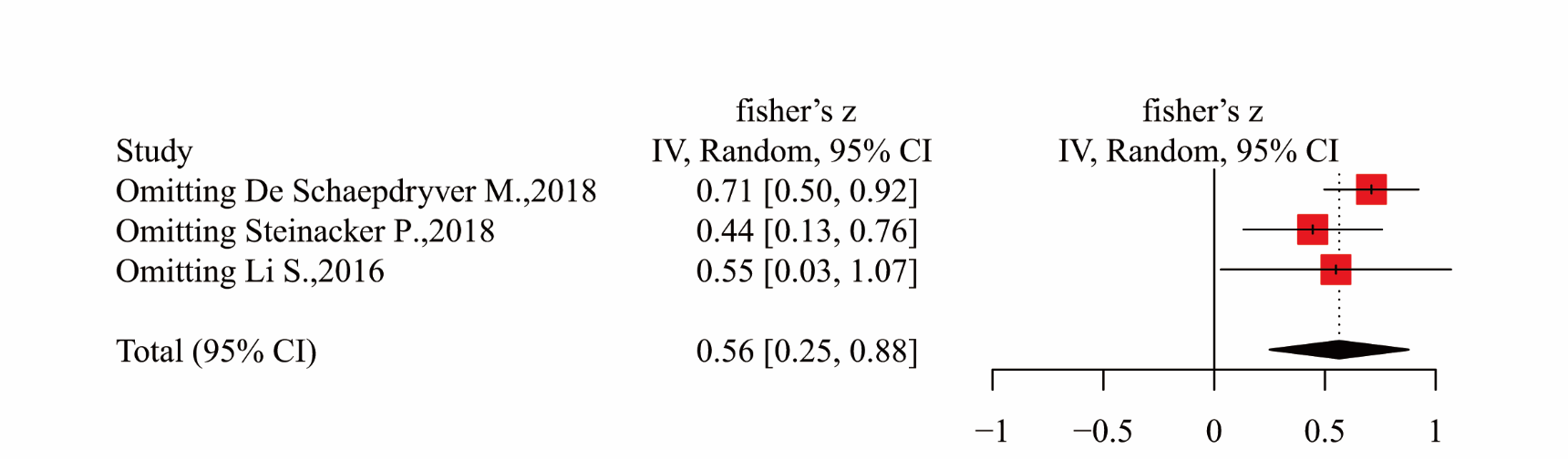


Figure 10. Sensitivity analysis diagram showing the relationship between pNfH levels and disease progression rate
